# Structural basis for the inhibition of poly(ADP-ribose) polymerases 1 and 2 by BMN 673, a potent inhibitor derived from dihydropyridophthalazinone

**DOI:** 10.1107/S2053230X14015088

**Published:** 2014-08-29

**Authors:** Mika Aoyagi-Scharber, Anna S. Gardberg, Bryan K. Yip, Bing Wang, Yuqiao Shen, Paul A. Fitzpatrick

**Affiliations:** a Research and Drug Discovery, BioMarin Pharmaceutical Inc., 105 Digital Drive, Novato, CA 94949, USA; b Emerald BioStructures, 7869 NE Day Road West, Bainbridge Island, WA 98110, USA

**Keywords:** poly(ADP-ribose) polymerase, PARP inhibitor, BMN 673, inhibitor design

## Abstract

Poly(ADP-ribose) polymerases 1 and 2 (PARP1 and PARP2), which are involved in DNA damage response, are targets of anticancer therapeutics. BMN 673 is a novel PARP1/2 inhibitor with substantially increased PARP-mediated tumor cytotoxicity and is now in later-stage clinical development for BRCA-deficient breast cancers. In co-crystal structures, BMN 673 is anchored to the nicotinamide-binding pocket *via* an extensive network of hydrogen-bonding and π-stacking interactions, including those mediated by active-site water molecules. The novel di-branched scaffold of BMN 673 extends the binding interactions towards the outer edges of the pocket, which exhibit the least sequence homology among PARP enzymes. The crystallographic structural analyses reported here therefore not only provide critical insights into the molecular basis for the exceptionally high potency of the clinical development candidate BMN 673, but also new opportunities for increasing inhibitor selectivity.

## Abstract 2

BMN 673, a novel PARP1/2 inhibitor in clinical development with substantial tumor cytotoxicity, forms extensive hydrogen-bonding and π-stacking in the nicotinamide pocket, with its unique disubstituted scaffold extending towards the less conserved edges of the pocket. These interactions might provide structural insight into the ability of BMN 673 to both inhibit catalysis and affect DNA-binding activity.

## 1. Introduction   

The family of poly(ADP-ribose) polymerase (PARP) enzymes plays a critical role in the detection and repair of DNA damage. The PARP enzymes share a common catalytic domain, in which an ADP-ribose moiety from NAD^+^ is transferred onto acceptor nuclear proteins, such as histones and PARP itself (Hassa & Hottiger, 2008 ▶). Poly(ADP-ribosylation) is a post-translational modification involved in various biological processes, including maintenance of genomic stability, transcriptional control, energy metabolism and cell death. Although PARP1, the most abundant member of the family, is reported to be responsible for the majority of cellular ADP-ribosyl­ation, at least some of its activity is mediated through heterodimerization with another member of the family, PARP2 (Amé *et al.*, 1999 ▶).

PARP1 and PARP2 are the most well studied members of the family. PARP1 is a 113 kDa protein consisting of three functional domains: an N-terminal DNA-binding domain, a central automodification domain and a C-terminal catalytic domain (de Murcia & Menissier de Murcia, 1994 ▶). A 62 kDa PARP2 enzyme, although structurally distinct, also has a DNA-binding domain and exhibits the highest degree of homology in the catalytic domain to that of PARP1 (Amé *et al.*, 1999 ▶). Extensive structural similarities of the catalytic domain of PARP2 to that of PARP1 were confirmed by the reported structures (Oliver *et al.*, 2004 ▶; Karlberg, Hammarstrom *et al.*, 2010 ▶). In both PARP1 and PARP2 the DNA-binding domain regulates enzymatic activity as a direct response to DNA damage (Hassa & Hottiger, 2008 ▶; Yélamos *et al.*, 2008 ▶).

The importance of PARP1 and PARP2 in DNA damage-response pathways has made these proteins attractive therapeutic targets for oncology (Rouleau *et al.*, 2010 ▶; Leung *et al.*, 2011 ▶; Ferraris, 2010 ▶). PARP1 and PARP2 inhibition could (i) enhance the cytotoxic effects of DNA-damaging agents by compromising the cancer-cell DNA-repair mechanisms and (ii) selectively kill tumors with inactivated homologous recombination DNA-repair pathways owing to deficiency in BRCA1/2 function. PARP1 has been an actively pursued drug-discovery target for the past three decades, leading to several promising PARP inhibitors in clinical development today (Kummar *et al.*, 2012 ▶; Ekblad *et al.*, 2013 ▶).

**Figure 1:**
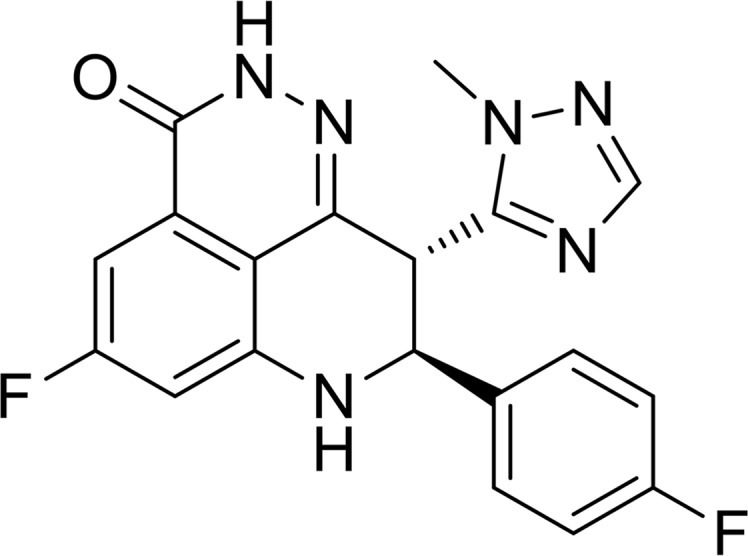
Chemical structure of BMN 673.

The majority of known PARP inhibitors are NAD^+^ competitive inhibitors. These inhibitors contain a carboxamide group that forms hydrogen bonds with Gly863 and Ser904, mimicking the binding mode of the nicotinamide group in the catalytic domain (Ferraris, 2010 ▶; Steffen *et al.*, 2013 ▶; Ekblad *et al.*, 2013 ▶; Papeo *et al.*, 2013 ▶). Built upon this conserved hydrogen-bond network, we have discovered and optimized a new chemical scaffold, leading to a highly potent PARP1/2 inhibitor, BMN 673 {(8*S*,9*R*)-5-fluoro-8-(4-fluorophenyl)-9-(1-methyl-1*H*-1,2,4-triazol-5-yl)-8,9-dihydro-2*H*-pyrido[4,3,2-de]­phthalazin-3(7*H*)-one; Fig. 1 ▶; Wang & Chu, 2011 ▶; Wang *et al.*, 2012 ▶}, with a reported IC_50_ value of 0.57 n*M* for PARP1 (Shen *et al.*, 2013 ▶). BMN 673, the most potent PARP inhibitor in clinical development, exhibits (i) high efficiency at killing tumor cells *in vitro*, possibly by effectively trapping PARP–DNA complexes (Shen *et al.*, 2013 ▶; Murai *et al.*, 2014 ▶), and (ii) impressive antitumor activity with limited toxicity in BRCA-deficient breast and ovarian cancer patients, and also early-stage clinical efficacy in a subset of small-cell lung cancer patients (Wainberg *et al.*, 2013 ▶). X-ray crystallographic analyses may reveal the molecular basis for the observed high potency and selectivity attainable by this new class of PARP inhibitors. Here, we present the structures of the catalytic domain of human PARP1 and PARP2 (catPARP1 and catPARP2) in complex with BMN 673, the most potent PARP inhibitor reported to date.

## 2. Materials and methods   

### 2.1. Protein and drug preparation   

A recombinant protein construct, catPARP1, with an N-terminal His_6_ tag, was produced in *Escherichia coli* BL21(DE3). The catPARP1 DNA insert, corresponding to the catalytic domain of human PARP1 (residues 662–1011), was subcloned into pET-28a (Novagen) *via* *Nde*I/*Xho*I restriction sites, resulting in the artificial N-terminal amino acids MGSSHHHHHHSSGLVPRGSHM. Upon reaching an optical density (OD_600_) of 0.5–0.8, catPARP1 protein expression was induced overnight at room temperature in Terrific Broth medium by adding 0.4 m*M* isopropyl β-d-1-thiogalactopyranoside (IPTG). Following cell lysis by sonication in 8.1 m*M* Na_2_HPO_4_, 1.5 m*M* KH_2_PO_4_, 138 m*M* NaCl, 2.7 m*M* KCl with EDTA-free protease-inhibitor cocktail (Thermo Scientific), the catPARP1 protein was first purified using a HiTrap Ni^2+^-chelating HP column (GE Healthcare) with a linear gradient elution of 10–250 m*M* imidazole in 20 m*M* NaPO_4_, 500 m*M* NaCl pH 7.5, followed by a HiPrep 26/60 Sephacryl S-300 HR gel-filtration column (GE Healthcare). The protein purity and ligand-binding activity (Shen *et al.*, 2013 ▶) were confirmed by SDS–PAGE and Biacore analyses, respectively. The purified catPARP1 in 25 m*M* Tris–HCl, 140 m*M* NaCl, 3 m*M* KCl pH 7.4 was stored at −80°C.

A recombinant catPARP2 protein, corresponding to the human PARP2 catalytic domain (residues 235–579) with an N-terminal His_6_ tag, was prepared as described in the literature (Karlberg, Hammarstrom *et al.*, 2010 ▶; Lehtiö *et al.*, 2009 ▶) with modifications. Briefly, catPARP2 protein expressed in *E. coli* T7 Express (New England BioLabs) was purified *via* three chromatographic steps: HiTrap Ni^2+^-chelating (GE Healthcare), POROS 50 HQ anion exchange (Applied Biosystems) and HiPrep 26/60 Sephacryl S-300 HR gel filtration (GE Healthcare). The catPARP2 protein was eluted from the Ni^2+^-chelating column by a linear gradient elution of 10–500 m*M* imidazole in 20 m*M* HEPES, 500 m*M* NaCl, 10% glycerol, 0.5 m*M* tris(2-carboxyethyl)phosphine (TCEP) pH 7.5. The POROS HQ column step was performed with a linear elution gradient of 25–500 m*M* NaCl in 25 m*M* Tris–HCl, 0.5 m*M* TCEP pH 7.8. The purified catPARP2 was stored in 20 m*M* HEPES, 300 m*M* NaCl, 10% glycerol, 1.5 m*M* TCEP at −80°C.

The synthesis of BMN 673 has been described elsewhere (Wang & Chu, 2011 ▶; Wang *et al.*, 2012 ▶).

### 2.2. Crystallization and data collection   

All crystallization experiments were performed by vapor diffusion at 16°C. Orthorhombic crystals of the catPARP1–BMN 673 complex were grown in the presence of 2.1 *M* ammonium sulfate, 0.1 *M* Tris–HCl pH 7.2–8.0, cryoprotected with 25%(*v*/*v*) glycerol and flash-cooled in liquid nitrogen. Diffraction data (Table 1 ▶) were collected on beamline 5.0.3 at the Advanced Light Source and were processed using *XDS* (Kabsch, 2010 ▶).

The catPARP2–BMN 673 complex was crystallized using 30%(*w*/*v*) PEG 3350, 0.25–0.33 *M* NaCl, 0.1 *M* Tris–HCl pH 8.5–9.1 as precipitant. Crystals were then cryoprotected in 25%(*v*/*v*) glycerol prior to flash-cooling in liquid nitrogen. Diffraction data were collected on beamline 7-1 at Stanford Synchrotron Radiation Lightsource and were processed (Table 1 ▶) as described above.

### 2.3. Structure determination and refinement   

The structure of the catPARP1–BMN 673 complex was solved by molecular replacement using published catPARP1 structures (PDB entries 1uk0 and 3l3m; Kinoshita *et al.*, 2004 ▶; Penning *et al.*, 2010 ▶) as search models using *Phaser* (McCoy *et al.*, 2007 ▶). The initial model of the catPARP1–BMN 673 complex, comprising four monomers in a crystallographic asymmetric unit, was refined through several cycles of manual model rebuilding in *Coot* (Emsley *et al.*, 2010 ▶) and refinement in *REFMAC*5 (Murshudov *et al.*, 2011 ▶) using TLS and noncrystallographic symmetry restraints. Statistics from data collection, final refinement and validation by *MolProbity* (Chen *et al.*, 2010 ▶) are summarized in Table 1 ▶.

The catPARP2–BMN 673 complex structure was solved and refined by the same methods with a few exceptions. A catPARP2 structure (PDB entry 3kcz; Karlberg, Hammarstrom *et al.*, 2010 ▶) was used as a template in molecular replacement. The catPARP2–BMN 673 crystals belonged to space group *P*1 and contained two monomers per asymmetric unit. Further details of data collection and structure refinement are provided in Table 1 ▶.

### 2.4. Structural analysis and visualization   

*MOE* (*Molecular Operating Environment*; Chemical Computing Group, Montreal, Canada), *Coot* (Emsley & Cowtan, 2004 ▶) and *PyMOL* (Schrödinger; http://www.pymol.org) were used for structural analyses and alignments and for generating figures.

## 3. Results   

### 3.1. Overall structures   

**Figure 2:**
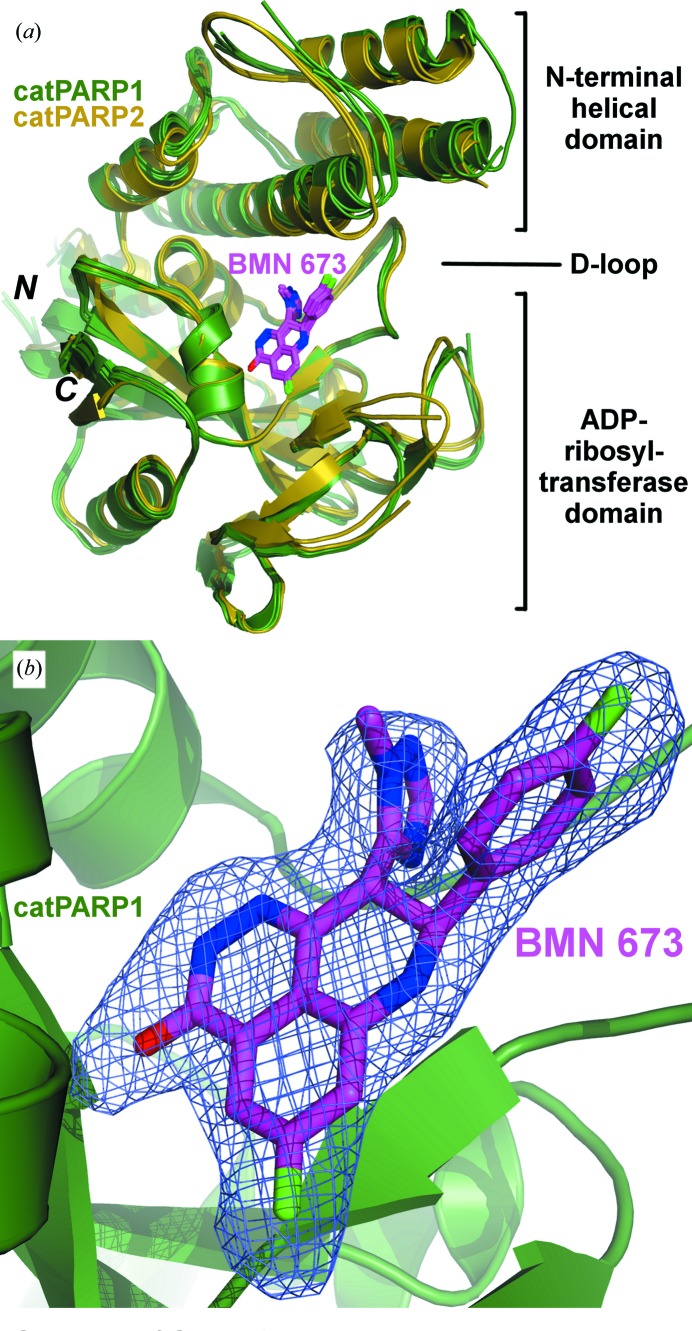
Co-crystal structures of catPARP1 and catPARP2 in complex with BMN 673. (*a*) Noncrystallographic symmetry-related molecules superimposed at the conserved pocket residues interacting with BMN 673. (*b*) *F*_o_ − *F*_c_ OMIT electron-density map (contoured at 2σ) of BMN 673 at the nicotinamide-binding site.

The crystal structures of catPARP1 bound to BMN 673 were solved and refined to 2.35 Å resolution (Table 1 ▶). As expected, these structures consist of an α-helical N-terminal domain and a mixed α/β C-terminal ADP-ribosyltransferase domain (Fig. 2 ▶ *a*), comparable to other catPARP1 structures described elsewhere (Kinoshita *et al.*, 2004 ▶; Iwashita *et al.*, 2005 ▶; Park *et al.*, 2010 ▶). The average pairwise root-mean-square deviation (r.m.s.d.) of the C^α^ atoms among these four monomers is 0.73 Å (Fig. 2 ▶ *a*). The pairwise C^α^ r.m.s.d. of these four copies with respect to the molecular-replacement search model (PDB entry 3l3m; Penning *et al.*, 2010 ▶) is also in the range 0.62–0.93 Å. Several catPARP1 regions, near residues Gln722–Ser725, Phe744–Pro749, Gly780–Lys787 and Lys1010–Thr1011, are disordered in the structure and associated with weak or absent electron density (Fig. 2 ▶ *a*). As observed in other catPARP1 structures (Ye *et al.*, 2013 ▶), a sulfate ion from the precipitant is bound at the putative pyrophosphate-binding site for the acceptor substrate poly(ADP-ribose) (Ruf *et al.*, 1998 ▶). Interestingly, our crystal structures unexpectedly show intermolecular disulfides formed by Cys845 residues from two different monomers (data not shown). The observed disulfide linkages are most likely to be experimental artifacts resulting from the nonreducing crystallization condition. More importantly, these disulfides are located on the protein surface and away (>20 Å) from the active site where BMN 673 is bound.

The co-crystal structure of catPARP2–BMN 673, solved and refined to 2.5 Å resolution (Table 1 ▶ and Fig. 2 ▶ *a*), exhibits a highly homologous overall structure to those of catPARP1/2 structures (Kinoshita *et al.*, 2004 ▶; Iwashita *et al.*, 2005 ▶; Park *et al.*, 2010 ▶; Karlberg, Hammarstrom *et al.*, 2010 ▶). An average pairwise r.m.s.d. (on C^α^ atoms) of 0.43 Å was calculated between our catPARP2 structures and the search model (PDB entry 3kcz; Karlberg, Hammarstrom *et al.*, 2010 ▶), comparable to the r.m.s.d. of 0.39 Å obtained between our two noncrystallographic symmetry-related molecules (Fig. 2 ▶ *a*). The disordered regions in the final catPARP2 models with weak electron density include residues Arg290–Gly295, Thr349–Glu355 and Asn548–Asp550 (Fig. 2 ▶ *a*). An average pairwise C^α^ r.m.s.d. of 1.15 Å signifies that the overall structural similarities between catPARP1 and catPARP2 are not perturbed by BMN 673 binding (Fig. 2 ▶ *a*).

### 3.2. Binding of BMN 673 to catPARP1   

BMN 673 binds in the catPARP1 nicotinamide-binding pocket *via* extensive hydrogen-bonding and π-stacking interactions. The well defined electron densities (Fig. 2 ▶ *b*) allowed unambiguous assignment of the orientation of BMN 673 in the pocket (Fig. 2 ▶ *a*), which consists of a base (Arg857–Gln875 in PARP1), walls (Ile895–Cys908), a lid (D-loop; Gly876–Gly894) (Wahlberg *et al.*, 2012 ▶; Steffen *et al.*, 2013 ▶) and a predicted catalytic residue, Glu988 (Ruf *et al.*, 1998 ▶). Several N-terminal helical bundle residues (αF; Ala755–Arg779) also line the outer edge of the binding pocket. The binding interactions of BMN 673 with catPARP1 can be broadly delineated into two parts: (i) conserved interactions formed at the pocket base with the nicotin­amide-like moiety of the inhibitor and (i) unique interactions formed at the outer edges of the pocket with the novel di-branched scaffold of the inhibitor.

**Figure 3:**
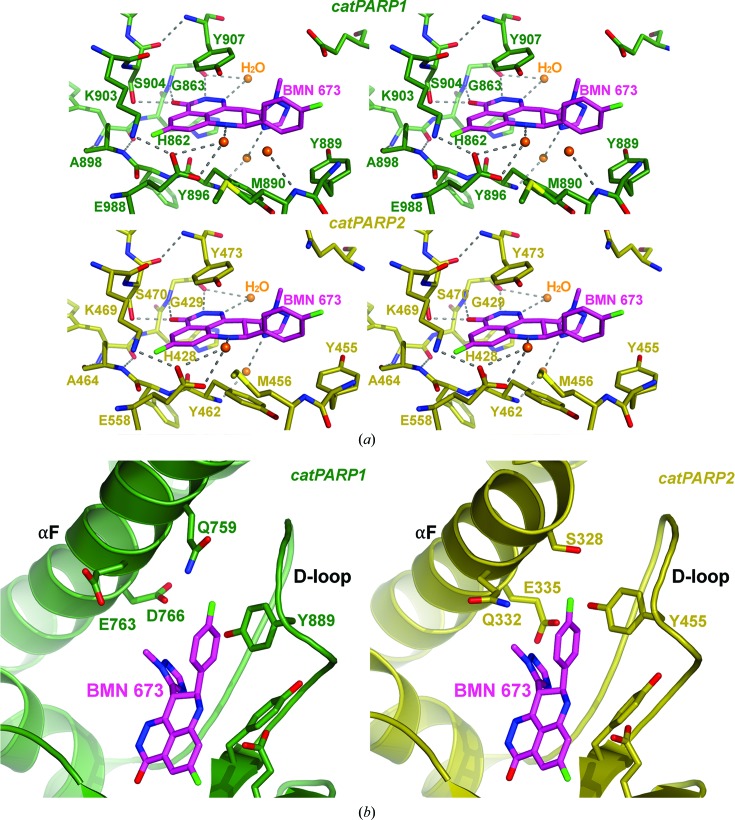
Binding mode of BMN 673. (*a*) Intricate network of hydrogen-bonding (dotted lines) and π-stacking interactions formed between BMN 673 and active-site residues (catPARP1–BMN 673 chain *D* and catPARP2–BMN 673 chain *A*). The novel disubstituted scaffold of BMN 673 leads to unique interactions with solvent molecules and extended pocket residues. (*b*) Binding interactions of BMN 673 at less conserved regions: the N-terminal helical domain (αF) and D-loop.

The core tricyclic group of BMN 673 is tethered to the base of the binding pocket *via* conserved stacking and hydrogen-bonding interactions. The cyclic amide moiety, commonly found in many known PARP inhibitors (Ferraris, 2010 ▶), forms hydrogen bonds with Gly863 backbone and Ser904 side-chain hydroxyl atoms (Fig. 3 ▶ *a*). A fluoro-substituted ring of the tricyclic core system is tightly packed against a small pocket formed by Ala898 and Lys903. The bound BMN 673 is surrounded with such aromatic residues as Tyr907, Tyr896 and His862; in particular, BMN 673 forms a π-stacking interaction with the nearby Tyr907 (∼3.6 Å; Fig. 3 ▶ *a*). Furthermore, the N atom (N7) from the unsaturated six-membered ring system is involved in a water-mediated hydrogen bond with Glu988 (Fig. 3 ▶ *a*), similar to the water-mediated interactions observed previously with a benzimidazole N atom (Penning *et al.*, 2008 ▶). In fact, these molecular interactions anchoring BMN 673 to the base of the NAD^+^-binding pocket represent well established binding features common to many PARP1/2 inhibitors described to date (Ferraris, 2010 ▶).

In addition to the relatively conserved inhibitor-binding interactions described above, BMN 673, with its unique stereospecific disubstituted [8*S*-(*p*-fluorophenyl), 9*R*-triazole] scaffold, forms several unprecedented interactions with ordered water molecules and residues at the outer edges of the binding pocket (Fig. 3 ▶ *a*). Firstly, the N atom (N4) in the triazole substituent is involved in a water-mediated hydrogen-bonding interaction to the backbone amide of Tyr896 (Fig. 3 ▶ *a*). This hydrogen-bond interaction appears to orient the tri­azole ring relative to the remaining inhibitor structure within the binding pocket. The triazole ring moiety also forms a H–π interaction with a water molecule, which is hydrogen-bonded to an N atom (N1) within the phthalazinone system of the inhibitor. The second substituent, an 8*S*-(*p*-fluorophenyl) group, forms π-stacking interactions with Tyr889 (Fig. 3 ▶ *a*). Furthermore, the fluorophenyl ring forms a H–π interaction with a nearby water molecule, which is in turn hydrogen-bonded to the Met890 backbone amide. The intricate network of hydrogen-bonding and π-stacking interactions between BMN 673, the water molecules and the extended binding-pocket residues explains the stereospecific inhibitory activity; BMN 673 is >250-fold more potent in inhibiting PARP1 than its enantiomer (Shen *et al.*, 2013 ▶). BMN 673 represents a new class of chiral PARP1/2 inhibitors that stereospecifically fit into the previously unexplored ligand-binding space near the lid of the NAD^+^-binding pocket.

### 3.3. Binding of BMN 673 to catPARP2   

As expected from overall and active-site structural similarities, BMN 673 binds the catPARP2 nicotinamide recognition site in a mode comparable to that described for the catPARP1 site (Fig. 3 ▶ *a*). Briefly, the amide core of BMN 673 is anchored to the base of the catPARP2 NAD^+^-binding pocket *via* the characteristic hydrogen-bonding interactions (Ferraris, 2010 ▶) involving Gly429 and Ser470 (Fig. 3 ▶ *a*). The fluoro-substituent on the tricyclic core of BMN 673 packs against Ala464 and Lys469 located on the walls surrounding the pocket. The bound BMN 673 is also sandwiched by the conserved aromatic residues Tyr473, Tyr462 and His428 in the pocket (Fig. 3 ▶ *a*). The ordered active-site water molecules mediate hydrogen-bonding and stacking interactions with the bound BMN 673. Finally, the unique stereospecific disubstituted moieties of BMN 673 at the 8 and 9 positions extend to the outer edge of the binding pocket, forming π-stacking interactions with Tyr455, as observed when bound to the catPARP1 active site (Fig. 3 ▶ *a*). Interestingly, the outer edges of the NAD^+^-binding pocket consist of the least conserved residues between catPARP2 and catPARP1.

### 3.4. Nonconserved residues in the BMN 673 binding site   

At the outer borders of the inhibitor-binding pocket, slight residue differences in the N-terminal helical bundle and D-loop at the active-site opening between the two PARP proteins are noteworthy (Fig. 3 ▶ *b*), especially when compared with the rest of the highly conserved active site. When bound to PARP2, a methyl group of the triazole moiety of BMN 673 points towards Gln332 on the N-terminal helical bundle; in PARP1, the same methyl group faces the highly mobile Glu763, which assumes various side-chain conformations among the noncrystallographic symmetry-related molecules. Also located on the N-terminal helical bundle, the PARP2-specific Ser328 is near the fluorophenyl substituent of BMN 673; in PARP1, the highly flexible Gln759 with multiple side-chain configurations occupies the corresponding position. In the PARP2 D-loop, Tyr455, which π-stacks with the fluorophenyl of BMN 673, is stabilized by direct hydrogen bonding to Glu335 on the N-terminal helical bundle (Fig. 3 ▶ *b*). On the PARP1 D-loop near the bound fluorophenyl group, a corresponding residue, Tyr889, is too distant to directly interact with the respective, but shorter, Asp766. Thus, the di-branched structure of BMN 673, extending to the least conserved outer active-site boundaries, potentially provides new opportunities for increasing inhibitor selectivity.

## 4. Discussion   

**Figure 4:**
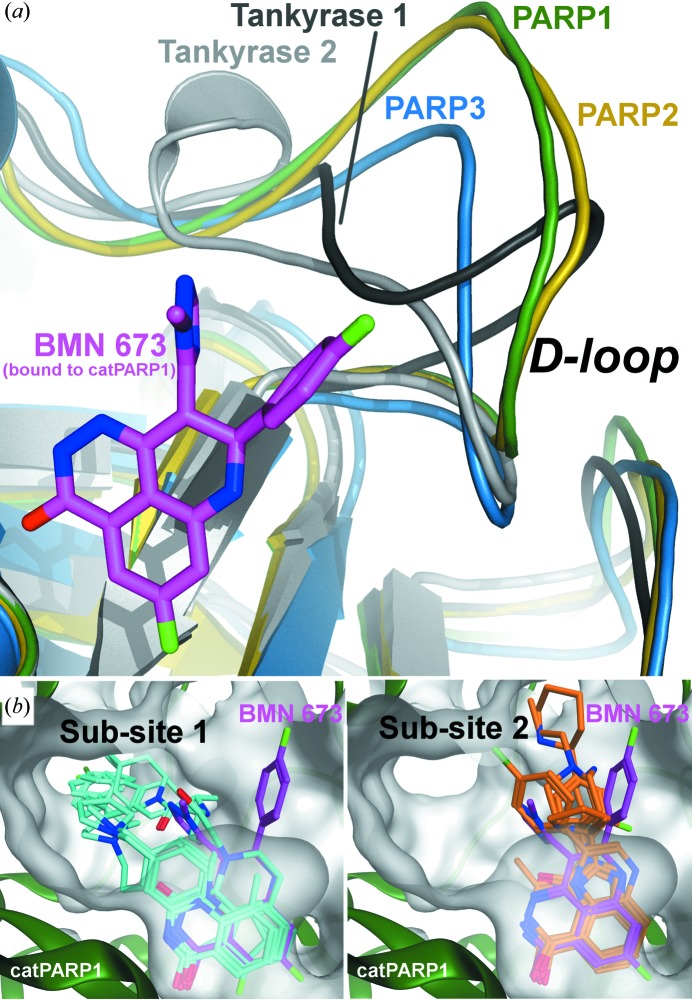
Binding of BMN 673 at the extended binding pocket. (*a*) Structural variability of the D-loop illustrated on superimposed crystallographic structures of PARP3 (PDB entry 3fhb; Lehtiö *et al.*, 2009 ▶), tankyrase 1 (2rf5; Lehtiö *et al.*, 2008 ▶) and tankyrase 2 (3kr7; Karlberg, Markova *et al.*, 2010 ▶), PARP1 and PARP2. (*b*) Unlike the other PARP1 inhibitors shown in cyan [PDB entries 1uk1 (Hattori *et al.*, 2004 ▶), 1uk0 (Kinoshita *et al.*, 2004 ▶), 3gjw (Miyashiro *et al.*, 2009 ▶), 4hhz (Ye *et al.*, 2013 ▶) and 4l6s (Gangloff *et al.*, 2013 ▶)] and orange [PDB entries 1wok (Iwashita *et al.*, 2005 ▶), 2rd6, 2rcw and 3gn7 (C. R. Park, unpublished work), 3l3m (Penning *et al.*, 2010 ▶), 3l3l (Gandhi *et al.*, 2010 ▶) and 4gv7 (Lindgren *et al.*, 2013 ▶)] which are directed towards sub-sites 1 and 2, a disubstituted BMN 673 molecule occupies a unique space within the extended NAD^+^-binding pocket.

Recent efforts in PARP inhibitor design have indeed centered on targeting sequence-variable and/or structure-variable regions outside the nicotinamide-binding pocket for improved specificity (Steffen *et al.*, 2013 ▶; Ekblad *et al.*, 2013 ▶). The aforementioned variable D-loop (Fig. 4 ▶ *a*) has been pursued as a druggable site for designing next-generation selective inhibitors (Andersson *et al.*, 2012 ▶). The aromatic D-loop residue, such as Tyr889 in PARP1 and Tyr455 in PARP2 (Fig. 3 ▶ *b*), which forms π-stacking interactions with the unique fluoro­phenyl group of BMN 673, is missing in PARP3 and tankyrases 1/2. The D-loop in PARP3 and tankyrases is also shorter and assumes distinct conformations (Fig. 4 ▶ *a*; Lehtiö *et al.*, 2009 ▶; Wahlberg *et al.*, 2012 ▶; Karlberg, Markova, *et al.*, 2010 ▶; Narwal *et al.*, 2012 ▶). Structural superposition indicates that the D-loop of PARP3 or tankyrases must undergo conformational changes in order to accommodate the fluorophenyl moiety of BMN 673 within the NAD^+^-binding pocket (Fig. 4 ▶ *a*). BMN 673, which fits in the unique binding space with structure and sequence diversity, therefore opens up new possibilities for selective inhibition of ADP-ribosyltransferase enzymes.

Targeting the noncatalytic function of PARP1/2 offers an alternative strategy for designing selective and potent PARP inhibitors. A crystal structure of essential PARP1 domains in complex with a DNA double-strand break revealed that inter-domain communication is mediated by the N-terminal α-helical bundle domain (Langelier *et al.*, 2012 ▶), towards which the triazole substituent of BMN 673 points (Fig. 3 ▶ *b*). Interestingly, BMN 673 is ∼100-fold more effective than other clinical PARP1/2 inhibitors at trapping PARP1/2 on DNA damage sites, a potentially key mechanism by which these inhibitors exert their cytotoxicity (Murai *et al.*, 2014 ▶). In fact, BMN 673 exhibits remarkable cytotoxicity in homologous recombination-deficient cells compared with other PARP1/2 inhibitors with a comparable ability to inhibit PARP catalysis (Shen *et al.*, 2013 ▶). The co-crystal structures of catPARP1 and catPARP2 in complex with BMN 673 reported here reveal that this highly potent inhibitor occupies a unique space within the extended NAD^+^-binding pocket (Fig. 4 ▶ *b*). Elucidating potential long-range structural effects that BMN 673, with its novel chiral disubstituted scaffold, might have on DNA binding and/or DNA damage-dependent allosteric regulation might aid in the development of new-generation PARP inhibitors with improved selectivity.

## Supplementary Material

PDB reference: catPARP1–BMN 673, 4pjt

PDB reference: catPARP2–BMN 673, 4pjv

## Figures and Tables

**Table 1:** Crystallographic data and refinement statistics Values in parentheses are for the outer shell.

	catPARP1–BMN 673 (PDB entry 4pjt)	catPARP2–BMN 673 (PDB entry 4pjv)
Data collection and processing		
Wavelength (Å)	0.9765	1.0970
Temperature (°C)	−173	−173
Detector	ADSC Quantum 315R	ADSC Quantum 315R
Crystal-to-detector distance (mm)	290	250
Rotation range per image (°)	1	1
Total rotation range (°)	180	180
Space group	*P*2_1_2_1_2_1_	*P*1
*a*, *b*, *c* (Å)	103.69, 108.15, 142.00	52.86, 57.74, 69.29
α, β, γ (°)	90.00, 90.00, 90.00	77.28, 79.99, 63.88
Resolution range (Å)	19.94–2.35 (2.40–2.35)	67.33–2.50 (2.56–2.50)
Total No. of reflections	459985	45124
No. of unique reflections	66890	22773
Completeness (%)	99.6 (99.4)	91.9 (91.3)
Multiplicity	6.9 (6.4)	2.0 (2.0)
〈*I*/σ(*I*)〉†	17.4 (3.8)	7.0 (1.8)
*R*_merge_ ‡	0.08 (0.48)	0.12 (0.46)
Refinement and validation		
Reflections, working set	63499	22773
Reflections, test set	3387	1150
Resolution range (Å)	19.94–2.35	67.33–2.50
*R*_work_ §/*R*_free_ ¶	0.190/0.228	0.214/0.287
No. of non-H atoms		
Protein	10190	5114
Ligands	205	74
Water	316	143
Mean *B* factors (Å^2^)		
Wilson *B* factor	43.4	25.7
Protein	42.9	21.3
Ligands	40.5	10.0
Water	36.2	10.9
R.m.s.d., bond lengths (Å)	0.012	0.011
R.m.s.d., bond angles (°)	1.461	1.467
Ramachandran plot		
Outliers (%)	0.1	0.0
Favored (%)	99.2	98.3

† Average signal-to-noise ratio.

‡ *R*_merge_ = 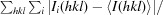 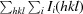.

§ *R*_work_ = 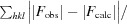 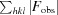, where *F*_obs_ and *F*_calc_ are the observed and calculated structure factors, respectively.

¶ 5% of the reflections were set aside randomly for *R*_free_ calculation.
